# Generalized linear mixed model for segregation distortion analysis

**DOI:** 10.1186/1471-2156-12-97

**Published:** 2011-11-11

**Authors:** Haimao Zhan, Shizhong Xu

**Affiliations:** 1 Department of Botany and Plant Sciences, University of California, Riverside, CA 92521

## Abstract

**Background:**

Segregation distortion is a phenomenon that the observed genotypic frequencies of a locus fall outside the expected Mendelian segregation ratio. The main cause of segregation distortion is viability selection on linked marker loci. These viability selection loci can be mapped using genome-wide marker information.

**Results:**

We developed a generalized linear mixed model (GLMM) under the liability model to jointly map all viability selection loci of the genome. Using a hierarchical generalized linear mixed model, we can handle the number of loci several times larger than the sample size. We used a dataset from an F_2 _mouse family derived from the cross of two inbred lines to test the model and detected a major segregation distortion locus contributing 75% of the variance of the underlying liability. Replicated simulation experiments confirm that the power of viability locus detection is high and the false positive rate is low.

**Conclusions:**

Not only can the method be used to detect segregation distortion loci, but also used for mapping quantitative trait loci of disease traits using case only data in humans and selected populations in plants and animals.

## Background

Segregation distortion refers to a phenomenon that the observed genotypic frequencies deviate significantly from the expected Mendelian frequencies [1]. Different populations have different Mendelian ratios, e.g., the typical Mendelian ratio for an F_2 _population is 1:2:1 for the three genotypes *A*_1_*A*_1_: *A*_1_*A*_2_: *A*_2_*A*_2_. Many reasons can explain the observed distortion [2-7]. The most promising explanation is viability selection on the distorted markers or loci linked to the markers [8]. In genetic mapping for quantitative traits, the basic assumption is Mendelian segregation [9]. Therefore, distorted markers are usually discarded prior to QTL mapping because people usually fear unexpected consequences of distorted markers on the results. In a recent study [10], we found that segregation distortion is not necessarily harmful to QTL mapping; rather, it can help in some circumstances. Consequently, we can incorporate segregation distortion into existing QTL mapping programs [11].

It appears that segregation distortion is common rather than rare. If segregation distortion is indeed caused by viability selection loci, these loci themselves are of interest because they may help to understand the mechanism of natural selection and evolution. Chi-square tests are commonly used to test segregation distortion. Fu and Ritland [12] and Lorieux et al. [13] developed maximum likelihood methods to map segregation distortion loci. The methods are interval mapping approaches in which one distortion locus is tested at a time. Vogl and Xu [14] used an MCMC implemented Bayesian algorithm to detect multiple segregation loci simultaneously. These methods are quite different from the usual QTL mapping procedures in quantitative trait genetic mapping. Luo and Xu [15] first developed an expectation and maximization (EM) algorithm for mapping viability selection loci. This method takes advantage of the well known EM algorithm in interval mapping. Recently, Luo et al. [16] developed a quantitative genetic model to map viability loci. The authors postulated a hidden underlying liability for each individual. The liability is an unobserved quantitative trait and natural selection acts on the liability. The method of Luo et al. [16] actually maps loci controlling the hidden liability (a quantitative trait). Therefore, methods of QTL mapping and viability locus mapping have been unified into the same framework of interval mapping. Both methods are called QTL mapping, but the traits mapped are different, the former maps observed quantitative traits and the latter maps unobserved liability.

The quantitative genetic model of Luo et al. [16] is an interval mapping approach. The state-of-the-art QTL mapping procedure is the Bayesian shrinkage method [17-19] because it simultaneously evaluates the entire genome. It is natural to extend the Bayeisan shrinkage method to map multiple viability loci. The Markov chain Monte Carlo (MCMC) algorithm is commonly used to implement the Bayesian method. Such a sampling based method is time consuming. A fast version of the Bayesian method is the empirical Bayesian method [20] where the variance components in the prior distributions of QTL effects are first estimated from the data and then used as the priors to estimate the QTL effects under the general Bayesian framework. This method is essentially the linear mixed model approach. When applied to discrete traits, the method is called the generalized linear mixed model [21,22].

Numerous algorithms have been developed to implement the generalized linear mixed model. The pseudo likelihood algorithm [23-25] appears to be the most popular one. The method requires a normal transformation of the original data point using the first step Newton-Raphson update. Once the data points are normally transformed, they are treated as normal quantitative phenotypes. The usual linear mixed model applies to the transformed data points. The difference between the Newton-Raphson transformation and the data transformation commonly seen in data analysis is that the Newton-Raphson transformation is a function of the data point and parameters while the usual data transformation is a function of the data point only. Therefore, the Newton-Raphson transformation is required for each cycle of the iteration process.

It is not clear how to use the pseudo likelihood approach to mapping viability loci because there is no phenotypic data point to transform. However, the method of McGilchrist [26] for generalized linear mixed model can be applied here. This method only requires a linear predictor, a likelihood and a prior distribution for each effect in the linear predictor. In this study, we used the McGilchrist's [26] method to perform parameter estimation.

## Method

### Liability model and viability selection

Let us define a continuous variable *y_j _*as the liability for individual *j*,

(1) $$y_{j}=X_{j}\beta +\overset{p}{\underset{k=1}{\sum }}Z_{jk}\gamma_{k}+\varepsilon_{j}$$

where *ε_j _*~ *N*(0,1) is a residual error with a standardized normal distribution. Other model effects are defined as follows. There may be some effects not related to genetics, such as age, location and other systematic effects, and these effects are captured by *β *and the design matrix *X*. There are *p *genetic loci each with an effect *γ_k _*for *k *= 1, ..., *p*. The value of *Z_jk _*is determined by the genotype of individual *j *at locus *k*. For example, an F_2 _individual derived from the cross of two inbred lines can take one of three genotypes, *A*_1_*A*_1_, *A*_1_*A*_2 _and *A*_2_*A*_2_. Under the additive genetic model, *Z_jk _*is defined as

(2) $$Z_{jk}=\left\{\begin{array}{c} +1\\ 0\\ -1 \end{array}\begin{array}{c} \text{for}\\ \text{for}\\ \text{for} \end{array}\begin{array}{c} A_{1}A_{1}\\ A_{1}A_{2}\\ A_{2}A_{2} \end{array}\right)$$

and *γ_k _*= *a_k _*is the additive genetic effect for locus *k*. Under the dominance effect model, the genetic effect for locus *k *is a 2 × 1 vector *γ_k _*= [*a_k _d_k_*]*^T^*, where *d_k _*is called the dominance effect. The corresponding *Z *variable is also a vector and defined as

(3) $$Z_{jk}=\left\{\begin{array}{c} H_{1}\\ H_{2}\\ H_{3} \end{array}\begin{array}{c} \text{for}\\ \text{for}\\ \text{for} \end{array}\begin{array}{c} A_{1}A_{1}\\ A_{1}A_{2}\\ A_{2}A_{2} \end{array}\right)$$

where *H_i _*is the *i*-th row of matrix *H*, as shown below,

(4) $$H=\left[\begin{array}{cc} +1 & -1\\ \;0 & +1\\ -1 & -1 \end{array}\right]$$

The liability *y_j _*is not observed but it determines the viability of individual *j*. It is assumed that individual *j *will survive if *y_j _*> 0 and die otherwise. Since we can only observe the surviving individuals, all individuals in the sample have liabilities greater than zero. This will cause the selected population to deviate from the expected Mendelian segregation ratio for loci responsible for viability selection and all loci linked to the viability loci. Although all individuals have survived, some may have a high liability and some may have a low liability, but all have a liability greater than zero. We now use the concept of penetrance to describe the survivability of an individual. Let

(5) $$\text{E}\left(y_{j}\right)=\eta_{j}=X_{j}\beta +\overset{p}{\underset{k=1}{\sum }}Z_{jk}\gamma_{k}$$

be the expectation of the unobserved liability (a linear predictor). We use the normal or the logistic function to model the probability of survival for individual *j*, i.e., Φ(*η_j_*) or logistic(*η_j_*) = exp(*η_j_*)/[1 + exp(*η_j_*)]. Conditional on the genotypes of all other loci, the penetrances for the three genotypes of locus *k *are defined as

(6) $$\left\{\begin{array}{c} \Phi \left(H_{1}\gamma_{k}+\eta_{j\left(-k\right)}\right)\\ \Phi \left(H_{2}\gamma_{k}+\eta_{j\left(-k\right)}\right)\\ \Phi \left(H_{3}\gamma_{k}+\eta_{j\left(-k\right)}\right) \end{array}\begin{array}{c} \text{for}\\ \text{for}\\ \text{for} \end{array}\begin{array}{c} G_{jk}=A_{1}A_{1}\\ G_{jk}=A_{1}A_{2}\\ G_{jk}=A_{2}A_{2} \end{array}\right)$$

where

(7) $$\eta_{j\left(-k\right)}=X_{j}\beta +\overset{p}{\underset{k^{\prime }\neq k}{\sum }}Z_{jk^{\prime }}\gamma_{k^{\prime }}$$

is the linear predictor excluding locus *k*. This model was first introduced by Luo et al. (2005) for single locus analysis, which does not include *η_j(-k) _*in equation (6). The data that allow us to estimate *γ_k _*is the genotype array for all individuals at locus *k*. Define

(8) $$w_{j}=\left[\begin{array}{ccc} w_{j\left(11\right)} & w_{j\left(12\right)} & w_{j\left(22\right)} \end{array}\right]$$

as a multivariate Bernoulli variable with three categories (i.e., a multinomial variable with sample size one). If individual *j *has a genotype *A*_1_*A*_1_, then *w*_*j*(11) _= 1 and *w*_*j*(12) _= *w*_*j*(22) _= 0. The probabilities of individual *j *taking the three genotypes are derived from the Bayes' theorem,

(9) $$\left\{\begin{array}{c} \pi_{j\left(11\right)}=\frac{1}{{\bar{\pi }}_{j}}\phi_{11}\Phi \left(H_{1}\gamma_{k}+\eta_{j\left(-k\right)}\right)\\ \pi_{j\left(12\right)}=\frac{1}{{\bar{\pi }}_{j}}\phi_{12}\Phi \left(H_{2}\gamma_{k}+\eta_{j\left(-k\right)}\right)\\ \pi_{j\left(22\right)}=\frac{1}{{\bar{\pi }}_{j}}\phi_{22}\Phi \left(H_{3}\gamma_{k}+\eta_{j\left(-k\right)}\right) \end{array}\right)$$

where

(10) $$\begin{array}{lll} {\bar{\pi }}_{j}= & \;\phi_{11}\Phi \left(H_{1}\gamma_{k}+\eta_{j\left(-k\right)}\right)\; & \text{(1)}\\ & +\phi_{12}\Phi \left(H_{2}\gamma_{k}+\eta_{j\left(-k\right)}\right)\; & \text{(2)}\\ & +\phi_{22}\Phi \left(H_{3}\gamma_{k}+\eta_{j\left(-k\right)}\right)\; & \text{(3)}\\ & \; & \text{(4)} \end{array}$$

is the mean of the three penetrances and

(11) $$\phi =\left[\begin{array}{ccc} \phi_{11} & \phi_{12} & \phi_{22} \end{array}\right]$$

is the expected Mendelian ratio. In an F_2 _population, the expected Mendelian ratio is $\phi =\left[\begin{array}{ccc} \frac{1}{4} & \frac{2}{4} & \frac{1}{4} \end{array}\right]$. Note that if *γ_k _*= 0, vector *π_j _*= [*π*_*j*(11) _*π*_*j*(12) _*π*_*j*(22)_] will be equivalent to the expected Mendelian ratio for every individual at the locus.

If there is no factor to be considered other than the markers, the term *X_j_β *should disappear here. This is different from the usual linear regression analysis where an intercept should always appear in the model. With the liability selection model, there is no intercept. We now assume only one co-factor to consider. The *X_j _*variable can be discrete or continuous, but the distribution in the unselected population must be known. In this study, we first assume that *X_j _*is discrete, say gender, a variable indicating the gender of individual *j *with *X_j _*= 1 representing male and *X_j _*= -1 representing female. In the unselected population, the sex ratio should be 1:1. If the population evaluated has a biased sex ratio, this means that the gender has an effect on the liability. We can estimate the gender effect *β *on the liability. Let $\varphi =\left[\begin{array}{cc} \varphi_{1} & \varphi_{2} \end{array}\right]=\left[\begin{array}{cc} \frac{1}{2} & \frac{1}{2} \end{array}\right]$ be the expected sex ratio (prior to the selection). Define *ξ*_*j*(1) _or *ξ*_*j*(2) _as the posterior probability that individual *j *is male or female, respectively. These posteriors are calculated using

(12) $$\left\{\begin{array}{c} \xi_{j\left(1\right)}=\frac{1}{{\bar{\xi }}_{j}}\varphi_{1}\Phi \left(\eta_{j\left(-\beta \right)}+\beta \right)\\ \xi_{j\left(2\right)}=\frac{1}{{\bar{\xi }}_{j}}\varphi_{2}\Phi \left(\eta_{j\left(-\beta \right)}-\beta \right) \end{array}\right)$$

where

(13) $${\bar{\xi }}_{j}=\varphi_{1}\Phi \left(\eta_{j\left(-\beta \right)}+\beta \right)+\varphi_{2}\Phi \left(\eta_{j\left(-\beta \right)}-\beta \right)$$

is the mean penetrance of the two genders and

(14) $$\eta_{j\left(-\beta \right)}=\overset{p}{\underset{k=1}{\sum }}Z_{jk}\gamma_{k}$$

is the linear predictor excluding the gender effect.

We now assume that *X_j _*is a continuous non-genetic effect, e.g., age. Let us assume that *X_j _*follows a normal distribution in the unselected population, i.e., *p*(*X_j_*) = *N*(*X_j_*|*μ*, *σ*^2^), where *μ *and *σ*^2 ^are known. Let *β *be the effect of *X_j _*on the liability. Define Φ(*X_j_β *+ *η*_*j*(-*β*)_) as the probability that individual *j *has survived the selection. The posterior probability is defined as

(15) $$\xi_{j}=\frac{1}{{\bar{\xi }}_{j}}N\left(X_{j}|\mu ,\sigma^{2}\right)\Phi \left(X_{j}\beta +\eta_{j\left(-\beta \right)}\right)$$

where

(16) $$\begin{array}{lll} {\bar{\xi }}_{j} & =\int_{-\infty }^{\infty }N\left(X_{j}|\mu ,\sigma^{2}\right)\Phi \left(X_{j}\beta +\eta_{j\left(-\beta \right)}\right)dX_{j}\; & \text{(1)}\\ \; & =\Phi \left[\left(\mu \beta +\eta_{j\left(-\beta \right)}\right)∕{\left(\sigma^{2}\beta^{2}+1\right)}^{1∕2}\right]\; & \text{(2)}\\ & \; & \text{(3)} \end{array}$$

Proof of this equation (16) is straightforward and thus given in the next paragraph.

Let *f*(*X_j_*) = *N*(*X_j_*|*μ*, *σ*^2^) be the normal density for variable *X_j _*with known *μ *and *σ*^2^. The following Lemma [27] is used to derive equation (16).

(17) $$\overset{+\infty }{\underset{-\infty }{\int }}f\left(X_{j}\right)\Phi \left(\frac{X_{j}-\xi }{\lambda }\right)dX_{j}=\Phi \left(\frac{\mu -\xi }{\sqrt{\sigma^{2}+\lambda^{2}}}\right)$$

Let us rewrite equation (16) as

(18) $$\begin{array}{lll} {\bar{\xi }}_{j} & =\overset{+\infty }{\underset{-\infty }{\int }}f\left(X_{j}\right)\Phi \left(X_{j}\beta +\eta_{j\left(-\beta \right)}\right)dX_{j}\; & \text{(1)}\\ & =\overset{+\infty }{\underset{-\infty }{\int }}f\left(X_{j}\right)\Phi \left[\frac{X_{j}-\left(-\eta_{j\left(-\beta \right)}∕\beta \right)}{1∕\beta }\right]dX_{j}\; & \text{(2)}\\ & \; & \text{(3)} \end{array}$$

Comparing equation (18) with equation (17), we can see that *ξ *= -*η*_*j*(-*β*)_/*β *and *λ*^2 ^= 1/*β*^2^. Substituting these into equation (17), we get

(19) $$\begin{array}{c} {\bar{\xi }}_{j}=\overset{+\infty }{\underset{-\infty }{\int }}f\left(X_{j}\right)\Phi \left[\frac{X_{j}-\left(-\eta_{j\left(-\beta \right)}∕\beta \right)}{1∕\beta }\right]dX_{j}\\ \;=\Phi \left(\frac{\mu -\left(-\eta_{j\left(-\beta \right)}∕\beta \right)}{\sqrt{\sigma^{2}+1∕\beta^{2}}}\right)\\ =\Phi \left(\frac{\mu \beta +\eta_{j\left(-\beta \right)}}{\sqrt{\sigma^{2}\beta^{2}+1}}\right) \end{array}$$

This concludes the derivation of equation (16) presented in the previous paragraph.

### Likelihood, prior and posterior

It is difficult (if not impossible) to construct the joint likelihood for all loci, but conditional on the effects and the genotypes of other loci, the likelihood for locus *k *can be derived based on the multivariate Bernoulli distribution, that is

(20) $$L\left(\gamma_{k}\right)=\overset{n}{\underset{j=1}{\sum }}\left[w_{j\left(11\right)}\ln\left(\pi_{j\left(11\right)}\right)+w_{j\left(12\right)}\ln\left(\pi_{j\left(12\right)}\right)+w_{j\left(22\right)}\ln\left(\pi_{j\left(22\right)}\right)\right]$$

The exact notation for this log likelihood should be *L*(*γ_k_*|*η*_(-*k*)_) because it is conditioned on the gender effect and effects of other loci. We use the simplified notation to improve the readability. Let us assign a normal prior to *γ_k_*, i.e.,

(21) $$p\left(\gamma_{k}\right)=N\left(\gamma_{k}|0,\Sigma_{k}^{}\right)$$

Furthermore, we assign a hierarchical prior to ∑*_k_*,

(22) $$p\left(\Sigma_{k}^{}\right)=\text{Inv - Wishart}\left(\Sigma_{k}^{}|\tau ,\omega \right)$$

where *τ *is the prior degree of freedom and *ω *is the prior scale matrix with the same dimension as ∑*_k_*. The reason for assigning these prior distributions is to handle a possible large number of loci involved in the model. Uniform prior for the gender effect is assumed. The log posterior (denoted by LogPost) is

(23) $$\text{LogPost}(\gamma_{k}\text{) }=L\left(\gamma_{k}\right)+\ln N\left(\gamma_{k}|0,\Sigma_{k}^{}\right)+\ln\left[\text{Inv - Wishart}\left(\Sigma_{k}^{}|\tau ,\omega \right)\right]$$

where a constant has been ignored.

For the sex effect (discrete co-factor), the likelihood for *β *conditional on *η*_*j*(-*β*) _is

(24) $$L\left(\beta \right)=\overset{n}{\underset{j=1}{\sum }}\left[\frac{1}{2}\left(X_{j}+1\right)\ln\left(\xi_{j\left(1\right)}\right)+\frac{1}{2}\left(1-X_{j}\right)\ln\left(\xi_{j\left(2\right)}\right)\right]$$

For the continuous co-factor, the log likelihood for parameter *β *can be written as

(25) $$\begin{array}{c} L\left(\beta \right)=\overset{n}{\underset{j=1}{\sum }}\ln\left(\xi_{j}\right)\\ =\overset{n}{\underset{j=1}{\sum }}\left\{\ln\Phi \left(X_{j}\beta +\eta_{j\left(-k\right)}\right)-\ln\Phi \left[\left(\mu \beta +\eta_{j\left(-\beta \right)}\right)∕{\left(\sigma^{2}\beta^{2}+1\right)}^{1∕2}\right]\right\} \end{array}$$

Prior distribution for the non-genetic effect is assumed to be uniform (uninformative prior) and thus only the likelihood is needed to find the posterior mode estimate of *β*.

### Posterior mode estimation

Due to the possible large number of parameters, we take a sequential approach to estimating the posterior mode parameters with one locus at a time. This approach is also called the coordinate descent algorithm. Once the parameters of all loci are updated, the sequence is repeated until a certain criterion of convergence is reached.

Let us define the first step of the Newton-Raphson iteration as

(26) $$\gamma_{k}^{\left(t+1\right)}=\gamma_{k}^{\left(t\right)}-{\left[\frac{\partial^{2}\text{LogPost}\left(\gamma_{k}\right)}{\partial \gamma_{k}\partial \gamma_{k}^{T}}\right]}^{-1}\left[\frac{\partial \text{LogPost}\left(\gamma_{k}\right)}{\partial \gamma_{k}}\right]$$

and denote the variance of this updated parameter by

(27) $$V_{k}^{}=-{\left[\frac{\partial^{2}\text{LogPost}\left(\gamma_{k}\right)}{\partial \gamma_{k}\partial \gamma_{k}^{T}}\right]}^{-1}$$

where the first and second partial derivatives are evaluated at $\gamma_{k}=\gamma_{k}^{\left(t\right)}$. The posterior mean and posterior variance matrix for *γ_k _*at iteration *t *are denoted by $\text{E}\left(\gamma_{k}\right)=\gamma_{k}^{\left(t+1\right)}$ and var(*γ_k_*) = *V_k_*, respectively. Since the posterior distribution of *γ_k _*is approximately multivariate normal (asymptotical theory), the posterior mean is identical to the posterior mode. The posterior of ∑*_k _*remains scaled inverse Wishart due to the conjugate property of the prior. Therefore, the posterior mode of ∑*_k _*is

(28) $$\Sigma_{k}^{\left(t+1\right)}=\frac{\text{E}\left(\gamma_{k}^{}\gamma_{k}^{T}\right)+\omega }{\left(\tau +1\right)+2+1}=\frac{\text{E}\left(\gamma_{k}^{}\right)\text{E}\left(\gamma_{k}^{T}\right)+\text{var}\;\left(\gamma_{k}^{}\right)+\omega }{\left(\tau +1\right)+2+1}$$

where τ + 1 is the degree of freedom for the inverse Wishart posterior and the number 2 represents the dimension of vector *γ_k_*.

The posterior mode estimation of *β *conditional on the effects of all loci is

(29) $$\beta^{\left(t+1\right)}=\beta^{\left(t\right)}-{\left[\frac{\partial^{2}L\left(\beta \right)}{\partial \beta \partial \beta^{T}}\right]}^{-1}\left[\frac{\partial L\left(\beta \right)}{\partial \beta }\right]$$

with an estimation error variance approximated by

(30) $$\text{var}\;\left(\beta \right)=-{\left[\frac{\partial^{2}L\left(\beta \right)}{\partial \beta \partial \beta^{T}}\right]}^{-1}$$

The iteration process of the posterior mode estimation is summarized as follows.

Step 0: Initialize all parameters.

Step 1: Update the non-genetic effect using equation (29).

Step 2: Update effect of marker *k *for *k *= 1, ⋯, *p *using equation (26).

Step 3: Update ∑*_k _*for *k *= 1, ⋯, *p *using equation (28).

Step 4: Repeat step 1 to step 3 until the iteration process converges.

### Genetic contribution from an individual locus

An obvious advantage of the liability model is that we are able to calculate the proportion of the liability variance contributed by each SDL, similar to the proportion of quantitative trait variance contributed by each QTL. Suppose that we have detected one SDL with both additive and dominance effects. The theoretical variances of the *Z *variables in an F_2 _population are 0.5 for the additive part and 1.0 for the dominance part. The reason is that the three genotypes are coded as +1, 0 and -1 for the additive *Z *and -1, 1 and -1 for the dominance *Z *[28]. Let *a_k _*and *d_k _*be the additive and dominance effects of this SDL. The genetic variance explained by this locus is

(31) $$V_{G}=\frac{1}{2}a_{k}^{2}+d_{k}^{2}$$

The residual variance of the liability is set at unity and thus the variance of the liability is

(32) $$V_{P}=V_{G}+1=\frac{1}{2}a_{k}^{2}+d_{k}^{2}+1$$

The broad sense heritability is defined as

(33) $$H=\frac{V_{G}}{V_{P}}=\frac{\frac{1}{2}a_{k}^{2}+d_{k}^{2}}{\frac{1}{2}a_{k}^{2}+d_{k}^{2}+1}$$

This is the proportion of the liability variance contributed by the *k*th SDL. Assuming that the multiple SDL are not closely linked, the overall contribution from all SDL is approximated by

(34) $$H=\frac{V_{G}}{V_{P}}=\frac{\sum_{k=1}^{p}\left(\frac{1}{2}a_{k}^{2}+d_{k}^{2}\right)}{\sum_{k=1}^{p}\left(\frac{1}{2}a_{k}^{2}+d_{k}^{2}\right)+1}$$

The liability model has unified QTL mapping and SDL mapping in the same framework of quantitative genetics.

## Results

### Mouse experiment

We used a published dataset of an F_2 _mouse experiment to demonstrate the application of the method. The dataset was published by Lan et al. [29] and is freely available from the internet. The mouse genome has 19 chromosomes (excluding the sex chromosome). The data contains 110 F_2 _*ob/ob *mice derived from the cross of two inbred lines (BT×BTBR) and 193 markers covering 1,800 cM of the entire mouse genome. The average marker distance was 9.35 cM per marker interval. We inserted one or more pseudo markers in intervals larger than 5 cM to make sure that the entire genome is evenly covered by (pseudo or true) markers with no intervals larger than 5 cM. The number of pseudo markers inserted was 273, resulting in a total of 466 markers (193 true and 273 pseudo markers). For the pseudo markers, the genotype indicator variable, *w_j _*= [*w*_*j*(11) _*W*_*j*(12) _*w*_*j*(22)_], is missing for every individual. In the data analysis, the missing variable was replaced by the conditional probability calculated using the multipoint method [30].

**Figure 1:**
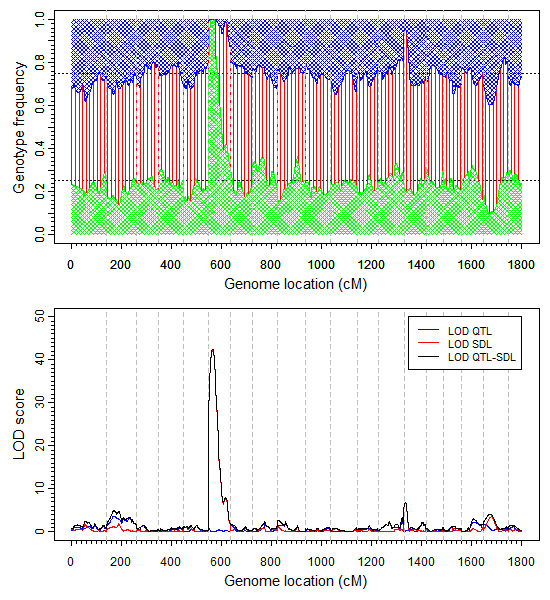
**Frequencies of the three genotypes and LOD score profiles of the mouse genome**. This is an F2 population derived from the cross of two inbred lines (BT×BTBR). (a) The top panel shows the frequencies of three genotypes with the blue, red and green patterns representing the *A*_1_*A*_1_, *A*_1_*A*_2 _and *A*_2_*A*_2 _genotypes, respectively. (b) The bottom panel shows the LOD score profiles for the mouse genome obtained from the interval mapping of segregation distortion. The profile in red (LOD SDL) represents the LOD score for segregation distortion. The curves in blue and black are the LOD scores for QTL of the 10 week body weight and joint testing of the QTL and segregation distortion.

The top panel of Figure 1 shows the frequencies of the three genotypes, *A*_1_*A*_1_, *A*_1_*A*_2 _and *A*_2_*A*_2_, plotted against the mouse genome. It is obvious that there is a severe distortion in the beginning of chromosome 6 where the population contains almost exclusively the *A*_2_*A*_2 _genotypes with *A*_1_*A*_1 _and *A*_1_*A*_2 _almost eliminated from the population. Chromosomes 14 and 18 also show mild segregation distortion. Interval mapping for segregation distortion using the QTL procedure in SAS [31] showed that the LOD score for chromosome 6 is 43.25 (see the bottom panel of Figure 1 for the LOD score profile obtained from the interval mapping analysis). The interval mapping procedure [31] is a separate analysis for each marker. With the interval mapping, the position with the highest LOD score (43.25) occurred at a pseudo marker (at position 15.69 cM) between the first true marker (D6Mit86, 0 cM) and the second true marker (D6Mit224, 30.4 cM) on chromosome 6. The estimated frequencies of this pseudo marker are 0.0000, 0.0001 and 0.9999 for the three genotypes (*A*_1_*A*_1_, *A*_1_*A*_2 _and *A*_2_*A*_2_), respectively.

**Figure 2:**
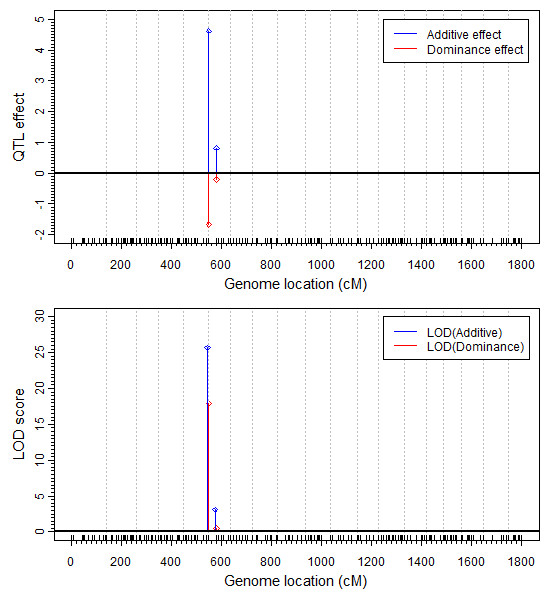
**QTL effects and LOD scores of the mouse genome estimated by GLMM**. The additive effect and the dominance effect are shown in the top panel and LOD score of additive effect and dominance effect are in the bottom panel. Additive effect and the LOD score profiles are colour coded in blue and the dominance effect and LOD score profiles are coded in red. Positions of the 193 true markers are indicated by the barcode like ticks on the horizontal axis. The critical value (95% of the LOD score generated under the null model) is 2.99, which is smaller than the observed LOD score of 25.0.

We used the generalized linear mixed model to analyze all the 466 markers (193 true and 273 pseudo) jointly. In the mouse data, among the 110 mice, 52 were male and 58 were female. Apparently, the sex ratio is not biased and thus sex appears to have no effect on the survivorship. However, we included the sex factor as a fixed effect in the model to test the robustness of our model. We expected that our model would detect no sex effect on the survivorship. The generalized linear mixed model had 466 × 2 + 1 = 933 model effects, including 466 additive effects, 466 dominance effects and one sex effect. This GLMM with 110 individuals was indeed able to handle such a large model (933 model effects). The hyper parameters used in the analysis was (*τ*, *ω*) = (0,0), equivalent to the Jeffrey's prior for the variance components. The estimated additive and dominance effects along with the corresponding LOD scores are depicted in Figure 2. One segregation distortion locus was detected on chromosome 6 (same as that of the interval mapping). The location of this distortion locus is right at the first marker of chromosome 6 (D6Mit86, 0 cM). The interval mapping approach described in the previous paragraph also detected a segregation distortion locus. However, the SDL detected by interval mapping was located halfway (15.69 cM) between markers D6Mit86 (0 cM) and D6Mit224 (30.4 cM) (see Figure 1 for the result of interval mapping). The GLMM analysis also showed some distortion for the second marker (D6Mit224, 30.4 cM), but the LOD score is only 3, barely significant. Therefore, we can safely ignore this locus due to linkage with the first marker. Let us go back to the first marker D6Mit86, the major SDL detected by the GLMM method. This segregation distortion locus is caused by both the additive and dominance effects. The estimated additive effect (± standard error) is $\hat{a}=4.6230\pm 0.4248$ while the estimated dominance effect (± standard error) is $\hat{d}=-1.6656\pm 0.1833$. The LOD scores are 25.69 and 17.92, respectively, for the additive and dominance effects. Simulation experiment under the null hypothesis (Mendelian segregation) showed that the 95% value of the null distribution of the LOD scores is 3.8, much smaller than the actual LOD score of 25.69. Therefore, we are very confident for this detected segregation distortion locus. As expected, the estimated sex effect is $\hat{\beta }=0.1969\pm 0.3002$ with a LOD score of 0.0934, smaller than 1.0255, the 95% value of the LOD score generated under the null model. Therefore, we can safely claim that the gender effect is insignificant.

In the GLMM analysis, the QTL effect has been interpreted as an effect on a hypothetical liability. The total variance of the liability is (see the Method section)

(35) $$\begin{array}{l} \sigma_{\text{Liability}}^{2}=0.5\times {\hat{a}}^{2}+{\hat{d}}^{2}+1\\ \;\;\;\;\;\;=0.5\times^{4.62302}+{\left(-1.6556\right)}^{2}+1\\ \;\;\;\;\;=14.4606 \end{array}$$

Therefore, the proportion of the liability variance explained by this segregation distortion locus is

(36) $$H=\frac{0.5\times {\hat{a}}^{2}+{\hat{d}}^{2}}{0.5\times {\hat{a}}^{2}+{\hat{d}}^{2}+1}=\frac{13.4606}{14.4606}=0.9308$$

which is also called the broad sense heritability. This single locus contributes approximately 93% of the liability variance. We can also calculate the expected frequencies of the three genotypic based on the estimated QTL effect. Let

(37) $$\begin{array}{l} \bar{\pi }=0.25\times \Phi (-\hat{a}-\hat{d})+0.5\times \Phi (\hat{d})+0.25\times \Phi (\hat{a}-\hat{d})\\ \;\;\;=0.0003878+0.0239483+0.25\\ \;\;\;=0.2743361 \end{array}$$

The expected frequencies for the three genotypes are

(38) $$\begin{array}{l} \pi_{11}=\frac{1}{\bar{\pi }}\times 0.25\times \Phi (-\hat{a}-\hat{d})=0.0014\\ \pi_{12}=\frac{1}{\bar{\pi }}\times 0.50\times \Phi (\hat{d})=0.0873\\ \pi_{22}=\frac{1}{\bar{\pi }}\times 0.25\times \Phi (\hat{a}-\hat{d})=0.9113 \end{array}$$

respectively, for *A*_1_*A*_1_, *A*_1_*A*_2 _and *A*_2_*A*_2_.

### Simulation experiment

**Figure 3:**
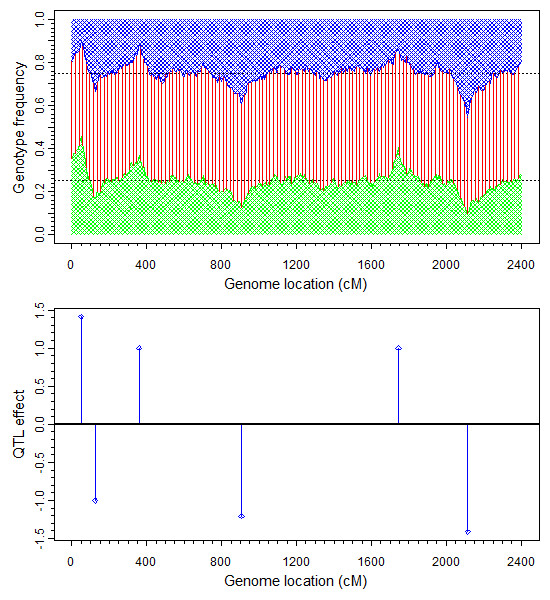
**Genotype frequencies and the true QTL effects for segregation distortion in the simulation experiment**. In the top panel, blue and green areas represent the frequencies of the two types of homozygote while the red area represents the frequency of the heterozygote. The true QTL effects are shown in the bottom panel.

We simulated a single chromosome with 2400 cM in length covered by 481 markers evenly placed on the genome with 5 cM per marker interval. The additive QTL effects of six markers were simulated with the true positions and true effects as presented in Figure 3 (bottom panel). Dominance effects were not simulated (zero values) although they were included in the data analysis. Frequencies of the three genotypes of a simulated F_2 _family with 500 individuals are also presented in Figure 3 (top panel). We also simulated two co-factors that influence the liability. The first co-factor was the sex effect coded as 1 for male and -1 for female with an effect value of *β*_1 _= 1.0. The second co-factor was a continuous variable with *μ *= 0 and *σ*^2 ^= 0.025. The effect of this co-factor on the liability was *β*_2 _= 1.0. The liability of each individual was generated using the linear model containing the two cofactors and the six QTL. An individual with a liability greater than 0 survived the selection, otherwise, it was eliminated. All the 500 individuals in the sample survived the selection. The simulated data were analyzed using the generalized linear mixed model with (*τ*, *ω*) = (0,0) as the hyper-parameter values.

**Figure 4:**
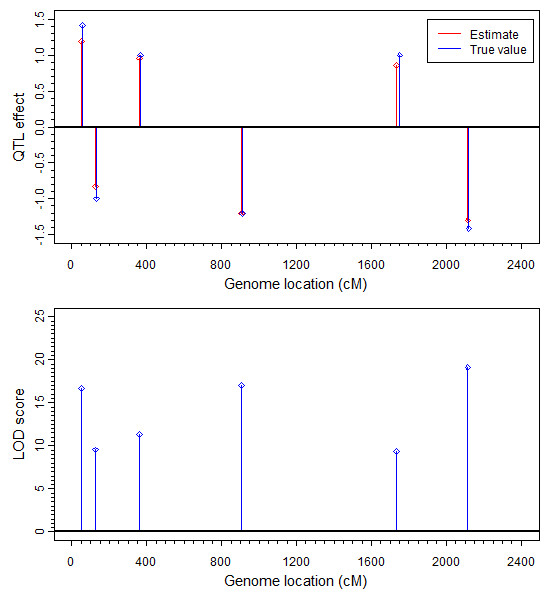
**Estimated additive QTL effects and the LOD scores for segregation distortion in the simulation experiment**. The additive QTL effects (top panel) and LOD scores for the additive effects (bottom panel) are estimated by GLMM. The true effect is colour coded in red and the estimated effect is coded in blue.

**Figure 5:**
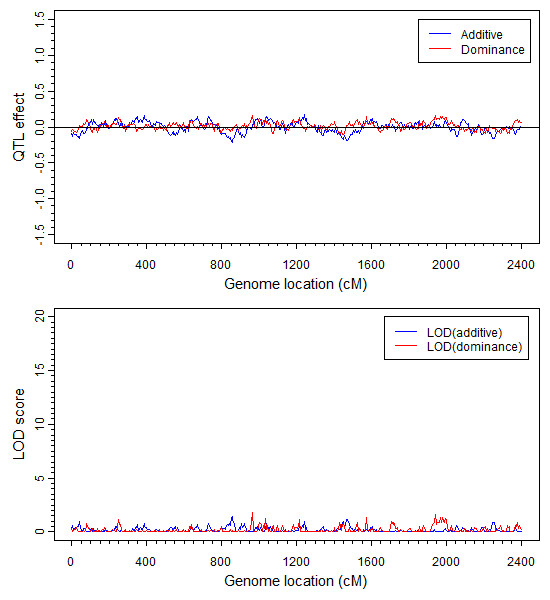
**The estimated QTL effects (top panel) and LOD scores (bottom panel) under the null model**. The data was simulated with no segregation distortion.

The estimated additive effects and the LOD scores are given in Figure 4. The estimated dominance effects and LOD scores were all near zero and thus not presented in the figure. Critical value of the LOD score generated from the null model was 2.99, which is smaller than the LOD score of each identified QTL. Therefore, all the six QTL have been identified by the method with no false positive identification. Figure 5 gives the estimated QTL effects and LOD scores for a dataset simulated under the null model. We can see that both the effects and the LOD scores are extremely small. The estimated QTL effects from simulation experiment (not the null model) are also presented in Table 1 along with the true values. Except QTL 5, all other QTL have been identified at the positions where they were simulated. QTL 5 was missed at the simulated position (1750 cM) but the effect was picked up at position 1735 cM, 15 cM away from the true position. The six QTL plus the two co-factors contributed 84.55% of the total variation of the liability and the estimated proportion was 82.74%, very close to the true proportion. The simulated data analysis demonstrates that the generalized linear mixed model successfully identified the simulated QTL and the two co-factors.

**Table 1:** Estimated parameters of the QTL identified by GLMM compared to true values in the simulation.

	True effect	True proportion	Estimate	StdErr	Position (cM)	LOD	Proportion
QTL 1	1.4135	0.1543	1.1905	0.1357	50	16.6828	0.1224
QTL 2	-0.9993	0.0771	-0.8296	0.1252	125	9.5271	0.0594
QTL 3	0.9993	0.0771	0.9605	0.1328	360	11.3536	0.0796
QTL 4	-1.2048	0.1121	-1.1991	0.1353	905	17.0304	0.1241
QTL 5	1.0000	0.0772	0.8593	0.1310	1735^a^	9.3347	0.0637
QTL 6	-1.41354	0.1543	-1.2959	0.1380	2115	19.1230	0.1450
Co-factor 1	1.0000	0.1545	1.0217	0.1020	--	21.7673	0.1803
Co-factor 2	1.0000	0.0386	1.1007	0.1809	--	8.0412	0.0523

		0.8455^b^					0.8272^c^

^a^1735 The true location is 1750 cM and the estimated location is 15 cM away from the true location.

^b^0.8455 This is the true (total) proportion of the liability variance contributed by the six QTL and the two co-factors.

^c^0.8272 This is the estimated (total) proportion of the liability variance contributed by the six QTL and the two co-factors.

This paragraph describes the result of 100 repeated simulations generated from the same set of parameters. This experiment allowed us to evaluate the power and false positive rate of QTL identification. The critical value for the LOD score was 2.99, which was generated empirically from multiple simulations under the null model (see the Method section). For each of the true QTL, if any marker with 15 cM away from the true QTL had a LOD score greater than 2.99, this QTL was declared as being detected. Since each marker interval was 5 cM, the 30 cM (15 cM left and right) coverage contained five markers (including the one with the true effect). If any marker more than 15 cM away from a simulated true QTL had a LOD score greater than 2.99, that marker was declared as a false positive. Results of the replicated simulation experiments are given in Table 2. The average estimated effects (QTL and co-factors) are consistently smaller than the true values due to the shrinkage nature of the estimation. The biases, however, are not too strong to affect the powers because all effects have been detected with very high powers (ranging from 71% to 100%). For the entire 100 replications, we only detected five false positives (positive markers that are 15 cM away from a true effect). The overall false positive rate is 5/[100 ×(481-5 × 6) = 0.0001111, extremely low. The number 481 in the denominator is the total number of markers, the number 6 is the number of markers with true effects and the number 5 is the number of markers in the window covering a true QTL.

**Table 2:** Average estimates of effects and powers of simulated QTL and co-factors from 100 replicated simulations.

	True	Estimate	StdEv	Power (%)
QTL 1	1.4135	1.1028	0.1329	99
QTL 2	-0.9993	-0.5964	0.1270	71
QTL 3	0.9993	0.7663	0.1474	91
QTL 4	-1.2048	-0.9858	0.1310	98
QTL 5	1.0000	0.7166	0.1375	87
QTL 6	-1.41354	-1.1977	0.1488	100
Co-factor 1	1.0000	0.9192	0.1299	100
Co-factor 2	1.0000	0.8894	0.1895	95

True - The true effects used to simulate the data.

Estimate - The average estimated effects obtained from 100 replicated simulation experiments.

StdEv - The standard deviation of effects from the 100 replications.

Power - The number of replicates in which the effect was detected out of 100 replicated samples

## Discussion and conclusions

Genome-wide segregation distortion is a common phenomenon in genetic mapping, but it is usually ignored. The main reason is the difficulty in joint estimation and tests of the segregation distortion loci. We formulated the problem as a typical quantitative genetics problem using a hypothetical liability to describe the fitness of each individual. Using a generalized linear mixed model, we were able to estimate and test genome-wide quantitative trait loci controlling the hidden liability. We used a mouse dataset to demonstrate the method and detected a major QTL for the liability that explains 93% of the liability variance. The simulated data experiment showed that the method can detect a QTL (e.g., the second QTL simulated) explaining 7.71% of the liability variation with 71% power. The method was implemented in a SAS/IML program. The code is posted on our website (http://www.statgen.ucr.edu) for general application. With this method and the program, genome-wide segregation distortion can be investigated routinely in future genetic data analysis.

As a Bayesian method, there are a rich array of prior distributions can be explored. In this study, we used the inverse Wishart as the prior distribution for the prior variance matrix of QTL effects. For the additive genetic model (one effect per locus), the inverse Wishart distribution becomes a scaled inverse Chi-square distribution. It is possible to use the exponential distribution (the Lasso prior) as an alternative prior [32]. Because the method uses multiple levels of prior choice, the model can also be called hierarchical generalized linear mixed model [24,33]. This study opens a new area in statistical genetics and further studies are expected to arise. For example, how to use the adaptive Lasso [34] to address this problem is entirely unknown and can be explored in the future.

A caveat of this method is the requirement of Mendelian segregation ratio (before the selection). For populations generated through line crossing experiments, Mendelian ratios are known. However, for uncontrolled populations, the theoretical Mendelian frequencies are not available. In this case, one needs to survey the unselected population to obtain the genotypic frequencies as the controlled "Mendelian segregation". If one can genotype both the selected and unselected individuals, one may simply use the case-control study and there is little reason to use this case-only study approach. In reality, genotyping individuals is much more costly than pooling the DNA of a sample of individuals. The cost effective approach is to genotype each individual in the surviving sample and genotype the pooled DNA sample for the unselected population because we only need the frequencies of genotypes (not the genotypes of individuals) in the unselected population. For the co-factors, we also need the expected frequencies of the co-factors in the unselected population. We examined the sex effect (discrete co-factor) and a normally distributed co-factor. The expected 1:1 sex ratio was used as the expected frequency. For the normal co-factor, we used the mean and variance of the co-factor used in the simulation (the true values) to construct the expected distribution. In reality, one needs to survey the entire population to obtain the expected distribution. For continuous variables deviating from normality, one may discretize a variable to a few groups. For example, age is a quantitative variable but one can arbitrarily divide individuals into a few age groups. This discretization will eliminate the restriction of normal distribution.

The method developed here can be applied to more broad situations beyond genetics without much modification. For example, if we know the joint distribution of *k *variables in a base (unselected) population and the joint distribution of the variables in a selected sample. We can simply test the difference between the two distributions to see which variables influence more on the selection. However, the pair-wise covariance may not allow us to make a precise decision on the importance of each variable. If two variables both influence the selection and they are highly correlated, the influence of one variable may be simply caused by the high correlation with the true causal variable. The proposed method here can help separate the true causality from the influence due to correlation.

QTL mapping is usually conducted in unselected populations. Individuals with undesired phenotypes must also be evaluated to obtain unbiased estimates of QTL effects. This is not a cost effective approach in breeding companies. Breeders wish to use only selected individuals to breed and keep no records for the unselected individuals. If we only evaluate the selected individuals, markers associated with the traits of interest will show distorted segregation. If the selection criterion is not well defined, for example, drought resistance, it is hard to map QTL. The segregation distortion loci are actually the QTL for drought resistance if one knows that there is no segregation distortion in the unselected population. The method developed here can be directly applied to mapping drought resistance QTL. Because we can perform QTL mapping using selected population, this approach may be called "mapping while selecting". For example, breeders may want to evaluate drought resistance of a family of recombinant inbred lines (RIL) by planting all seeds in a harsh drought environment. Eventually all plants die except the ones with strong resistance of drought. Breeders may have no records of the plants eliminated, but they can still perform QTL mapping for this trait (drought resistance) using all plants that have survived the selection. Other stress related traits can also be mapped using this approach, e.g., pest and salinity resistances.

In human genetics, case-control study is a common approach for mapping disease loci. In situations where there are no records for the control but the case, this case-only study may benefit from the new method. For example, one may easily get patient data from hospitals but hardly has individual records for the entire population. QTL mapping for the disease trait is still possible if we have the population records (frequencies) of genotypes in the entire population.

In summary, we developed a hierarchical generalized linear mixed model to map QTL for liability. This is a new approach to genetic mapping. It incorporates a seemingly different problem (segregation distortion) into the same QTL mapping framework for quantitative traits. Statistically, it shows that the generalized linear mixed model can be applied to situations where there are no phenotypic records; one only needs a likelihood function, a linear predictor and a prior distribution to infer the posterior mode estimation of the model effects.

## Competing interests

The authors declare that they have no competing interests.

## Authors' contributions

HZ conducted the actual work in terms of programming and data analysis. SX proposed the idea, oversaw the project and wrote the manuscript. All authors read and approved the final manuscript.
